# Active IgG4-related disease with bone marrow involvement: a report of 2 cases and case-based review

**DOI:** 10.1186/s40001-022-00643-w

**Published:** 2022-02-02

**Authors:** Shiyi Liu, Hui Wang, Tao Su

**Affiliations:** 1 Renal Division, Department of Medicine, Peking University First Hospital, Beijing, China; 2 Institute of Nephrology, Peking University, Beijing, China; 3 Renal Division, Department of Medicine, Wangjing Hospital, China Academy of Chinese Medical Sciences, Beijing, China; 4 Laboratory of Electron Microscopy, Peking University First Hospital, Beijing, China

**Keywords:** IgG4-related disease, Bone marrow, Hematologic, Leukopenia, Thrombocytopenia, ^18^F-FDG-PET/CT

## Abstract

IgG4-related disease (IgG4-RD) is a systemic fibro-inflammatory disease, histopathologically characterized by dense lymphoplasmacytic infiltration rich in IgG4-positive plasma cells in affected organs. Classic hematologic presentations including lymphadenopathy, eosinophilia and polyclonal hypergammaglobulinemia are common, whereas bone marrow involvement of IgG4-RD is rarely reported. Here we present two patients of multi-organ IgG4-RD with bone marrow involvement, one on bone marrow biopsy, and the other on PET/CT. Presentations of other organ involvement included biopsy-proven IgG4-related tubulointerstitial nephritis, lymphadenopathies, submaxillary glands, arteritis, asthma, dysosmia, and constitutional symptoms. Bone marrow involvement was initially suspected due to leukopenia, anemia and thrombocytopenia in case#1, and was finally confirmed by histological evidence of significant IgG4-positive plasma cells infiltration in bone marrow. In case#2, we incidentally observed high uptakes of multi-bone marrow on ^18^F-FDG-PET/CT, with the maximum SUV value similar to that of the kidneys, submaxillary glands and hilar, mediastinal lymph nodes by ^18^F-FDG-PET/CT. Symptoms and all the hematologic presentation improved rapidly in both patients after steroids initiation. These two cases illustrate the rare bone marrow involvement in active IgG4-RD accompanied by other hematologic syndromes. The significance of disease pathogenesis is worthy of further study.

## Introduction

IgG4-related disease (IgG4-RD) is a fibro-inflammatory condition recognized for decades, characterized by multi-organ enlargement with histopathological dense lymphoplasmacytic infiltration rich in IgG4-positive plasma cells, storiform fibrosis and obliterative phlebitis. IgG4-RD was first reported in patients with autoimmune pancreatitis (AIP) and it was gradually recognized as a systemic immune disease over the following years [1, 2]. Usually, most patients with IgG4-RD present with polyclonal hypergammaglobulinemia, which is typically caused by elevation of serum IgG4 [3]. Additionally, elevated serum IgG4 is much more common in Asian patients than patients of European descent [4, 5]. Nearly all the tissues can be involved, especially the pancreas, salivary glands, biliary duct, liver and kidneys. Hematologic manifestations of IgG4-RD have also been recognized, such as lymphadenopathy [6]. But case reports of bone marrow (BM) involvement were rare.

Here, we present two patients of IgG4-RD with bone marrow involvement. One patient had significant IgG4-positive plasma cells infiltration in bone marrow aspiration and biopsy samples. In the other patient, hematologic involvement was detected by 18 fluorodeoxyglucose positron emission tomography (^18^F-FDG-PET/CT), showing high uptakes in multi-bone marrow. These reports suggest the possibility of hematologic pathogenesis of IgG4-RD.

## Materials and methods

### Case presentation

**Fig.1:**
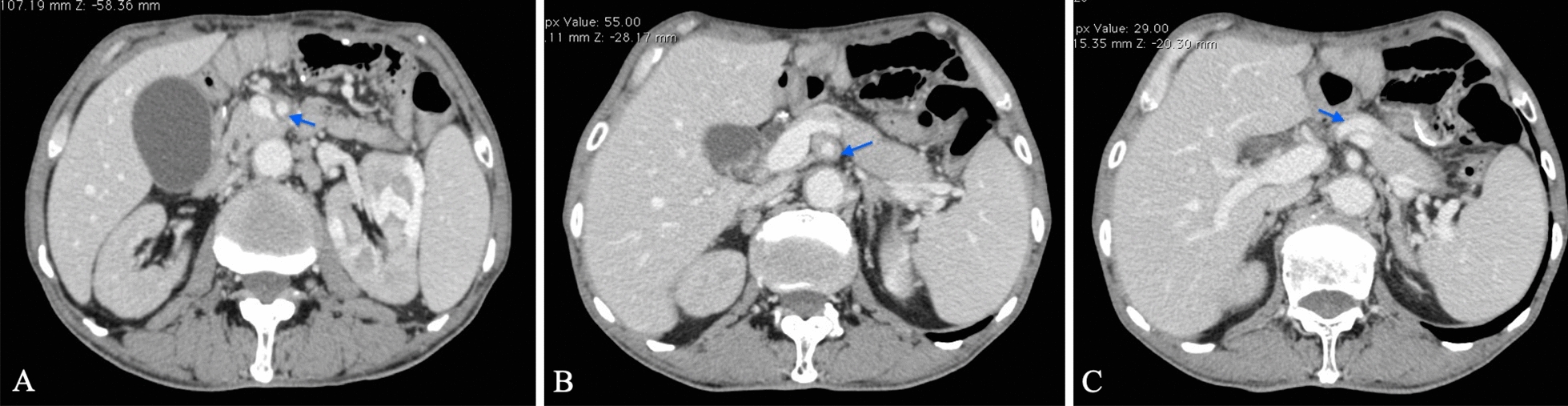
Enhanced computed tomography scan of patient 1: diffuse aortic wall thickening in the superior mesenteric (**A**, arrow in blue), abdominal aorta (**B**) and splenic arteries (**C**)

Case 1 was a 68-year-old Chinese male who complained of upper abdominal pain with aggravation of renal function. He had a 2-year history of repeated upper abdominal pain suspected as “ileus” and suffered frequent attacks during the last 9 months. The patient developed acute kidney injury initially as serum creatinine (Scr) was insidiously elevated from 270 to a peak of 410 μmol/L, and went back to normal range after supportive treatment. However, symptoms relapsed 2 weeks before his hospital admission to Peking University First Hospital. The abdominal enhanced-CT scan revealed swelling kidneys with renal parenchyma patchy filling defects, and dilated small bowel loops. Thickening of the abdominal aorta, superior mesenteric and splenic artery walls could also be recognized (Fig. 1).

**Fig.2:**
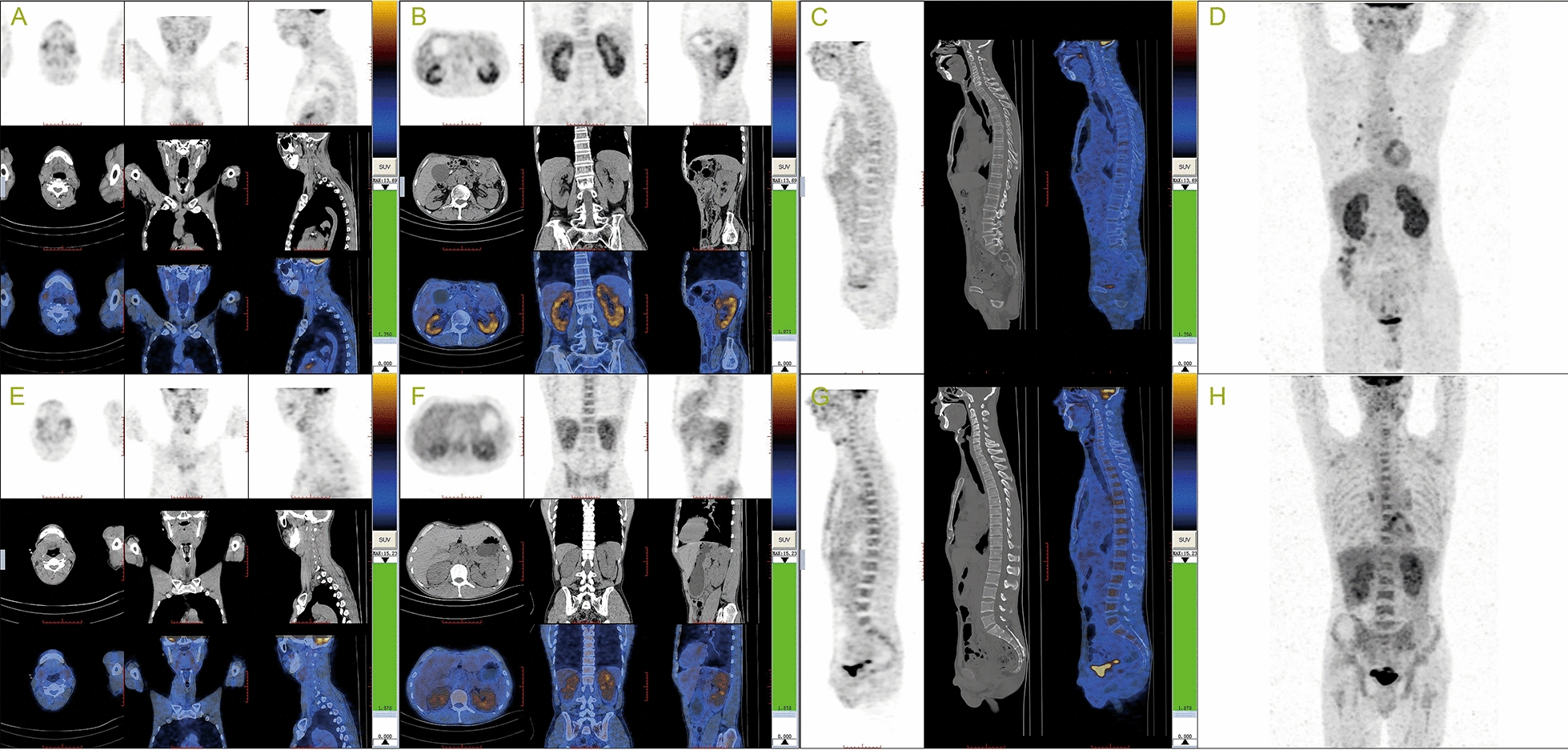
Positron emission tomography–computed tomography scans of the two patients. Patient 1, high uptakes in bilateral submandibular glands (**A**, SUV^max^ 3.0), kidneys (**B**, SUV^max^ 5.0), hilar and mediastinal lymph nodes on MIP images (**D**); absence of bone marrow (**C**). Patient 2, high uptakes in bilateral submandibular glands (**E**, SUV^max^ 2.9), kidneys (**F**, SUV^max^ 3.7), bone marrow (**G**, SUV^max^ 3.6), hilar and mediastinal lymph nodes on MIP images (**H**)

Laboratory testing details on admission are shown in Table 1. The patient had mild leukopenia, anemia and thrombocytopenia. The Scr was 326 μmol/L in the presence of mild proteinuria. The serum protein electrophoresis showed polyclonal hypergammaglobulinemia (IgG 29.56 g/L, IgG4 11.7 g/L), and no monoclonal band was detected. ^18^F-FDG-PET/CT scan showed high uptakes in bilateral submaxillary glands, kidneys, hilar and mediastinal lymph nodes (Table 1, Fig. 2). In contrast, abdominal aorta, superior mesenteric, splenic arteries and bone marrow showed no hypermetabolic lesions.

**Table 1:** Clinical, laboratory and radiological findings in two IgG4-RD patients

	Case 1	Case 2
IgG4-related clinical symptoms	AKI, AKD on CKD	AKD
	Ileus	Asthma, rhinitis
	Fatigue	Fatigue, weight loss
IgG4-RD involved organs, identified by		
Clinical presentation/enhanced-CT/US (measured by US, cm^3^)	Kidneys (13.0 × 6.3 × 5.8/12.2 × 5.9 × 4.5)	Kidneys (13.0 × 7.5 × 6.4/13.3 × 6.6 × 5.7)
	Submaxillary glands	Submaxillary glands
	Abdominal aortitis	–
	Superior mesenteric, splenic arteritis	–
Pathology	Kidney (right)	Kidney (right)
	Bone marrow	ND
FDG-PET/CT (SUV max)	Kidneys (5.0)	Kidneys (3.7)
	Submaxillary glands (3.0)	Submaxillary glands (2.9)
	Hilar, mediastinal lymph nodes (4.0)	Hilar, mediastinal lymph node (3.5)
	–	Bone marrow (3.6)
Number of involved sites	6	6
Laboratory data		
WBC (3.5–9.5 × 10^9^/L)	3.4	4.2
NE (40.0–75.0%)	60.8	54.8
LY (20.0–50.0%)	27.2	18.8
EO (0.4–8.0%)	4.0	15.0
Platelet count (125–350 × 10^9^/L)	110	173
Hemoglobin (130–175 g/L)	108	122
Total protein (65–85 g/L)	73.1	99.5
Albumin (40–55 g/L)	34.3	36.7
BUN (1.8–7.1 mmol/L)	17.27	7.2
Creatinine (44–133 μmol/L)	326	146
Amylase (35–135 IU/L)	219	ND
C3 (0.6–1.5 g/L)	0.519	0.486
C4 (0.12–0.36 g/L)	0.15	0.113
IgG (7.23–16.85 g/L)	29.56	64.77
IgG1 (4.90–11.40 g/L)	8.35	12.60
IgG2 (1.69–7.86 g/L)	8.01	3.57
IgG3 (0.11–0.86 g/L)	1.50	1.40
IgG4 (0.03–2.01 g/L)	11.70	47.20
IgA (0.69–3.82 g/L)	2.33	1.01
IgM (0.63–2.77 g/L)	0.50	0.57
IgE (< 100 kU/L)	ND	1761
hs-CRP (0.0–3.0 mg/L)	8.56	9.99
ESR (0–15 mm/h)	52	92
Urinary protein (0–0.15 g/day)	0.53	0.41
ANA	1:3200	< 1:100
dsDNA (IU/mL)	262	< 1:10
Lymphocytes T CD4^ + ^(34.0–70.0%)	ND	38.26
Lymphocytes T CD8^ + ^(14.0–41.0%)	ND	57.46

*AKI* acute kidney injury, *AKD* acute kidney disease; *ND*, no data; *US* ultrasonography

**Fig. 3:**
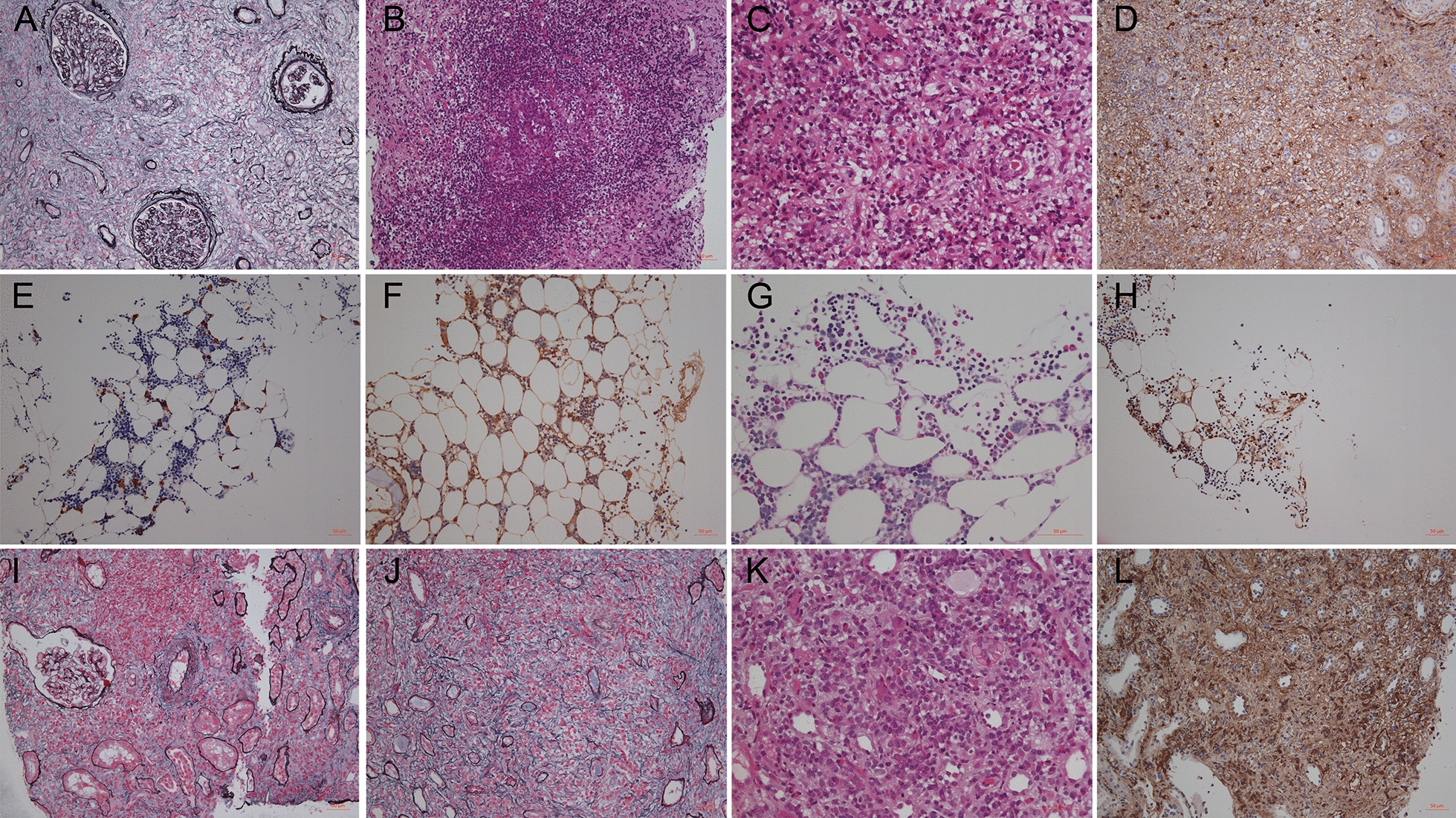
Histopathological findings of kidneys and the bone marrow. Patient 1, renal fibrosis was remarkable, “storiform” fibrosis existed in the local area (**A**, H&E stain, 200 × ). The formation of an ectopic germinal center-like structure was seen in the local interstitium (**B**, H&E stain, 200 × ). In local interstitium, diffuse inflammatory cells infiltration was seen in the interstitium, mainly lymphocytes, plasma cells and easily observed eosinophils (**C**, H&E stain, 400 × ) on hematoxylin and eosin staining. IgG4-positive plasma cells counted more than 10 cells per high power field on immunohistochemical staining for IgG4 (**D**, IgG4 stain, 200 × ). The bone marrow proliferation of patient 1 was active, no obvious abnormality was found in granulocyte and lymphocyte lines from BM samples. However, we found CD138 (**E**, CD138 stain, 200 × ) and IgG (**F**, IgG stain, 200 × ) positive plasma cells were scattered in the bone marrow interstitium with some eosinophils (**G**, eosinophils stain, 400 × ), and 50% of these IgG-positive plasma cells were positive for IgG4 (**H**, IgG4 stain, 200 × ). The plasma cells presented weak positive of both kappa and lambda light chain. Patient 2 had massively infiltrated inflammation cells in the renal interstitium including lymphocytes, plasma cells and eosinophils (**I**, H&E stain, 200 × ; **K**, H&E stain, 400 × ). The large area of interstitial fibrosis with local typical storiform pattern could be observed on hematoxylin and eosin staining (**J**, H&E stain, 200 × ). IgG4-positive plasma cells counted more than 10 cells per high power field on immunohistochemical staining for IgG4 (**L**, IgG4 stain, 200 × )

The patient underwent a right kidney biopsy. Renal pathology disclosed ischemic sclerotic glomeruli (19/28, 67.9%) with mild mesangial proliferation and typical IgG4-related tubulointerstitial nephritis. The ratio of IgG4/IgG was 65% (Fig. 3) in immunohistologic staining. Renal fibrosis was remarkable and “storiform” fibrosis existed in the local area. The formation of an ectopic germinal center was found. He also had a posterior superior iliac spine bone marrow examination because of unexplained leukopenia and thrombocytopenia. Analysis of bone marrow could exclude hematologic malignancies. Bone marrow proliferation was active. Granulocyte lines accounted for 65.5%, with the ratio of granulocyte to erythroid at 2.5:1. Five megakaryocytes could be seen in the whole BM smear (2.0 × 0.5 cm), and no plasma cell was observed in BM aspirate. In BM biopsy, we found CD138 and IgG-positive plasma cells were scattered in the BM interstitium with some eosinophils, and 50% of these IgG-positive plasma cells were also positive for IgG4 (IgG 40/HP, IgG4 20/HP). The plasma cells presented weak positives for kappa and lambda light chains (Fig. 3). Together, the diagnosis of IgG4-RD was confirmed with six sites involved and constitutional symptoms. The patient was prescribed prednisolone 40 mg/day. Symptoms were relieved soon after prednisolone initiation. The peripheral blood cell count returned to normal. Scr declined to 240 μmol/L one month after hospital discharge and remained at 180 μmol/L during the subsequent year.

Case 2 was a 38-year-old male referred to the nephrology clinic for investigation of renal insufficiency as his serum creatinine was 146 μmol/L. He presented severe fatigue, dysosmia and conspicuous weight loss of 11 pounds during the last 2 months. He had a history of anaphylactic rhinitis and bronchial asthma. On admission, blood pressure was 150/90 mmHg, physical examination revealed bilateral submandibular gland swelling with left supraclavicular lymphadenopathy, and these were later confirmed by ultrasonography and FDG-PET/CT scan.

Laboratory tests are summarized in Table 1. It showed obvious eosinophilia with normal WBCs and platelet count. There was a remarkable hyperglobulinemia (IgG 64.8 g/L, IgG4 47.2 g/L, IgE 1761 kU/L). No monoclonal immunoglobulin was detected by immunofixation electrophoresis from serum and urine. ^18^F-FDG-PET/CT showed high uptakes in bilateral submandibular glands, kidneys and bilateral scapula, spine, sternum, ribs, pelvis, and upper femur (Fig. 2), indicating signs of IgG4-relating lesions including bone marrow. Kidney pathological findings showed that all glomeruli were mildly changed without immune deposits on immunofluorescence. There were massively infiltrated inflammation cells in the renal interstitium including lymphocytes, plasma cells and eosinophils, as well as diffuse interstitial fibrosis accompanied by local storiform fibrosis. IgG4 to IgG-positive plasma cells were 40% (Fig. 3). A diagnosis of IgG4-related tubulointerstitial nephritis was made.

The patient was initiated on prednisolone combined with plasmapheresis. Four weeks later when he was dismissed from hospital, his IgG4 decreased to 1.49 g/L and Scr to 108 μmol/L. Two doses of Rituximab 375 mg/m^2^ with a 4-week interval were infused. The patient currently remained stable for 2 years.

### Literature search

A systematic review of the literature was done of IgG4-RD with involvement of bone marrow published before September 2021 on PubMed/MEDLINE. Our search included a combination of keywords such as, “IgG4-related disease”, “bone marrow”, “thrombocytopenia”, “leukopenia” and “anemia”. Our search was limited to English articles including original articles, review articles and case reports. Reported hematologic presentations which were irrelevant to IgG4-RD or did not contain detailed information were excluded.

## Discussion

IgG4-RD is a systemic disease characterized by largely consistent pathomorphological manifestations across a wide range of organ systems. We call it a pathologically fibro-inflammatory pattern with enlarged organs or tissues. With the increasing number of reports and research in this field, clinicians have gradually become familiar with this syndrome. IgG4-related bone marrow presentation has rarely been mentioned before [7], since fibrosis and obliterative phlebitis is typically not seen, resulting in insensitivity to diagnosis [8]. Kamisawa et al*.* firstly described histologic feature of IgG4-positive plasma cell infiltration of the BM in two patients with AIP in 2003 [9]. Moreover, in a case report of isolated IgG4-RKD with kappa light chain deposit, BM biopsy revealed around 20% of plasma cell infiltration without monoclonal paraprotein [8]. As reported in published studies, BM involvement of IgG4-RD usually presented pancytopenia, accompanied by other hematologic manifestations, including lymphadenopathy, eosinophilia and polyclonal hypergammaglobulinemia [6], and was finally confirmed by subsequent BM pathology. In addition, a case of IgG4-RD primarily presenting as multiple vertebral BM lesions on MR imaging and ^18^F-FDG-PET/CT [10], was finally identified IgG4-related BM involvement by pathology. A recently published PET/CT research by Schmidkonz et al. [11] declared that lesions with high uptake in ^18^F-FDG-PET/CT showed dense lymphoplasmacytic infiltration of IgG4 cells in histology; and that fibroblast activation protein imaging could disentangle inflammatory from fibrotic disease activity. In the current study, we reported one patient (#1) showing IgG4-dominated plasma cells infiltration in BM, with no hypermetabolic lesion found in FDG-PET/CT. For another patient (#2), we identified elevated FDG uptake lesions involving multi-bone marrow in the context of the active IgG4-RD. Reported cases of definite IgG4-related bone marrow involvement were rare as listed in Table 2. A recent flow cytometric analysis found different immunophenotyping of atypical plasma cells in peripheral blood (accounting for 58.3%, expressing CD19^low^/CD38^+^/CD138^dim^/CD56^−^/CD45^+^) and in the BM (accounting for 64.3%, expressing CD19^low^/CD38^+^/CD138^+^/CD56^+^/CD45^low^) [12]. As an adhesion molecule, the difference in CD56 expression might be related to the migration ability to the circulating system. However, the exact explanation was unclear.

**Table 2:** Literature review of bone marrow involvement in IgG4-RD

No.	Author	Age range (years)	Race, gender	Extra-hematologic organs involved in IgG4-RD	Presentation of hematologic involvement	Detection of bone marrow involvement	Treatment	Prognosis
1	Kim [12]	60–65	Korean, M	Kidney: IgG4-related tubulointerstitial nephritis Skin: rash (possible associated with eosinophilia)	Multiple lymphadenopathies; leukocytosis, thrombocytopenia, marked eosinophilia (51%), and rare nucleated red blood cells (1/100 WBCs)	FDG-PET/CT: hyperactive BM; BM biopsy: 20.4% eosinophils; 16.8% immature plasma cells; increased numbers of CD138-positive plasma cells; IgG4/IgG-positive plasma cell ratio = 0.88 PB: 58.3% of plasma cells were atypical (low CD19 expression with CD38^ + /^CD138^dim^/CD56^ − ^/CD45^ + ^	High-dose steroids	Symptoms improved
2	Ichiki [7]	60–65	Japanese, F	Submandibular gland swelling	Anemia, IgG4-related lymphadenopathies by lymph node biopsy	FDG-PET/CT: uptakes in multiple bones and immunohistochemically proven by BM biopsy BM biopsy: > 50% of CD138-positive plasma cells were IgG4 positive	Steroids 0.5 mg/kg	Anemia and submandibular gland swelling improved
3	van den Elshout-den Uyl [10]	75–80	Netherland, M	Fatigue, increased exhaustion after physical exercise, weight loss, and night sweats	Anemia and leucocytosis	MRI: showed multiple hypodense bone lesions FDG-PET/CT: FDG-avid bone lesions along the spinal cord BM biopsy: < 10% plasma cells in pre-existing bone marrow, but around 25% plasma cells were present in the fibrotic lesion	Prednisolone starting dose 40 mg/day	Symptoms improved, as well as serum hemoglobin and ESR
4	Tarte [33]	40–45	African-American, M	Kidneys (TIN, imaging abnormalities), lungs, liver, and small intestine (imaging abnormalities)	Lymphadenopathies (biopsy-proven), anemia and thrombocytopenia	BM biopsy: mild plasmacytosis (10–15% of total cellularity). a subset of plasma cells exhibited specificity for IgG4 + stain (< 20% of total IgG + plasma cells)	Prednisone 60 mg/day, Rituximab	Symptoms improved
5		65–70	Chinese, M	Kidneys (IgG4-TIN, imaging abnormalities), submaxillary glands, arteritis	Leukopenia, anemia and thrombocytopenia, lymphadenopathies	BM biopsy: 50% of CD138-positive plasma cells were IgG4-positive, scattered eosinophils	Prednisone 40 mg/day	Symptoms improved
6		35–40	Chinese, M	Kidneys (IgG4-TIN), submandibular gland	Marked eosinophilia (15%), lymphadenopathies	FDG-PET/CT: hyperactive BM	Prednisone 50 mg/day, Rituximab	Symptoms improved

5 and 6 are our presented cases

*PB* peripheral blood, *BM* bone marrow, *MRI* magnetic resonance imaging, *TIN* tubulointerstitial nephritis

Lymphadenopathy is the common presentation in IgG4-RD. Involved lymph nodes typically have > 100 IgG4 + plasma cells/HPF with an IgG4/IgG ratio > 40%. In addition, some pathologists have noticed ectopic germinal centers formed in IgG4-RD affected tissues and their possible relationship with dense deposits along tubular basement membrane [13, 14], as findings showed in our case#1. Peripheral lymph tissues mainly include lymph nodes and mucosa-associated lymphoid tissue where circulating plasmablasts, an indicator of IgG4-RD disease activity, further differentiate and proliferate to form mature plasma cells and produce antibodies [15]. And tissue-resident antibody-secreting plasma cells are the major interstitial infiltrating inflammation cells. As reported in many clinical studies, serum IgG4 level correlates with lymph node involvement and the number of affected organs. IgG4-RD may also be related to the occurrence of hematologic tumors. The elevated level of plasmablasts from continuously stimulated B lymphocytes, which reflects the IgG4-RD inflammation severity, might lead to B lymphocyte lymphoma. A previous study reported a special case [16] of extranodal marginal zone B cell lymphoma in the background of IgG4-RD, with malignant tumor cells existing in the presence of non-neoplastic cells. Moreover, in the differentiation process, plasmablasts are possibly transformed into tumorous plasma cells producing numerous monoclonal IgG4 molecules. This supports the comment that once a high level of serum IgG4 was found, monoclonal IgG4 must be excluded. There have been case reports of IgG4 myeloma being mistaken for IgG4-RD [17–19], as well as IgG4 POEMS syndrome [20]. These provide proof that the IgG4 molecule is likely a reactive product of immune dysregulation rather than the primary etiologic mediator in IgG4-RD.

Eosinophilia and tissue eosinophils infiltration are common in IgG4-RD. Eosinophilic angiocentric fibrosis is now part of IgG4-RD, usually affecting orbits and the upper respiratory tract. In the current report, renal eosinophils aggregation was obvious in both patients, even with an absence of eosinophilia as in case#1. A previous study presented similar findings that eosinophils infiltration was evident in 30.8% of IgG4-RD patients compared with 9.5% in non-IgG4 tubulointerstitial nephritis [21]. Regulation of IgG4 production is dependent on Th2 cells and is therefore linked to IgE response and eosinophilia, indicating an underlying inflammation. However, the role of eosinophils in immune-mediated IgG4-RD is not completely understood. In some recently published studies [21–23], the significance of eosinophils to renal fibrosis progression has been focused on. And in animal models, the loss of eosinophil peroxidase could reduce renal fibrosis [23]. All these evidence suggest that eosinophils may represent the inherent characteristic of the immune response in IgG4-RD [24, 25]. Sometimes, IgG4-RD with eosinophilia needs to be differentiated from lymphocyte variant hypereosinophilic syndrome and autoimmune lymphoproliferative syndrome, because both diseases could present eosinophilia and involve the lymph nodes [26, 27].

Disease diagnosis and monitoring based on clinical syndromes and general imaging findings are currently required criteria. Pathological examination of involved tissue remains irreplaceable in the diagnosis of IgG4-RD. While in the absence of pathological evidence, it is difficult to accurately distinguish inflammatory from fibrotic activity in human immune-mediated disease in vivo. ^18^F-FDG-PET/CT has been considered a promising diagnostic tool to aid IgG4-RD diagnosis in recent years. As a whole-body imaging technique, it is reported to detect more lesions in 71.4% of IgG4-RD patients than the conventional imaging methods, providing a more comprehensive evaluation of disease distribution as an entity [27]. In another reported study, ^18^F-FDG-PET/CT had a sensitivity of 85.7% and specificity of 66.1% for diagnosing IgG4-RD [28]. The intensity of ^18^F-FDG uptake in IgG4-RD changes along with disease remission and relapse [29]. Although the difference in cutoff SUV values between IgG4-RD and early-stage malignancies is not significant, there are still recognizable characteristics [27], such as the distribution characteristics of hypermetabolic lesions, contributing to a more accurate judgment. Patient #1 showed prominent IgG4-positive plasma cells infiltration in the bone marrow and arteritis on enhanced-CT, but ^18^F-FDG-PET/CT did not show high uptake in both sites. It was speculated that ^18^F-FDG might not be able to visualize chronic fibrotic lesions. At present, more novel PET probes have been exploited to better target fibrotic lesions (i.e., ^68^FAPI) [11] or to detect early changes in lymphoid mass in systemic autoimmunity (i.e., ^18^F-fluoroarabinofuranosyl cytosine) [30]. This would allow evidence for a more accurate evaluation of immunosuppressive therapy.

Steroids are still the mainstay for IgG4-RD therapy. However, its long-term use is not recommended. B cells depletion strategy is superior to steroids, especially for refractory and recurrent cases. Rituximab has shown a response rate of 97% in one prospective trial [31] and a lower relapse rate [32]. In case#1, steroids helped to reach partial remission of renal injury and systemic inflammation. His kidney function finally remained at chronic kidney disease stage 4. Oppositely, case #2 was young and diagnosed with early onset of disease, plasma exchange combined with steroids plus Rituximab led to complete remission.

## Conclusion

These two cases illustrate the rare bone marrow involvement in active IgG4-RD accompanied by other hematologic syndromes. The significance of disease pathogenesis is worthy of further study.

## Data Availability

The patients were regularly followed up and the clinical data are traceable. The datasets used and analyzed during the current study are available from the corresponding author on reasonable request.
